# Introducing *Juncigena alexandrina* sp. nov. (Ascomycota, Juncigenaceae) from the Mediterranean coast of Egypt, based on morphology and multi-locus phylogeny

**DOI:** 10.1186/s12866-026-04825-y

**Published:** 2026-03-07

**Authors:** Mahmoud Saadeldin Bakhit

**Affiliations:** Department of Botany and Microbiology, Faculty of Science, Sohag University, Sohag, 82524 Egypt

**Keywords:** *Phragmites australis*, Sexual morph, Sordariomycetes, Taxonomy, Torpedosporales

## Abstract

A novel marine fungal species, *Juncigena alexandrina* is described and illustrated from a decaying stem of *Phragmites australis* collected from El-Shatby beach of the Mediterranean Sea in Alexandria Governorate, Egypt. Phylogenetic analyses of the combined SSU, ITS, and LSU sequence data placed the new taxon within *Juncigena* as a phylogenetically distinct species within a clade containing *J*. *adarca* and *J*. *fruticosae*. Morphologically, the new fungus is characterized by having semi-immersed to erumpent ascomata with a long ostiolar neck covered with branched hairs, 8-spored, cylindrical to clavate asci, and hyaline, fusiform, 3-septate ascospores. It differs from the only known sexual morph in the genus, *Juncigena adarca*, by having wider, semi-immersed to erumpent ascomata with an ostiolar neck covered by long, branched hairs, wider asci and larger ascospores. *Juncigena fruticosae* is known only from its asexual stage. The discovery of *Juncigena alexandrina* expands current understanding of species diversity, morphological variation, and substrate associations within the genus *Juncigena*.

**Supplementary Information:**

The online version contains supplementary material available at 10.1186/s12866-026-04825-y.

## Introduction

*Juncigenaceae* was introduced by Jones et al. [1] to accommodate *Juncigena* Kohlm., Volkm.-Kohlm. & O.E. Erikss., as the type genus and to include the genera *Fulvocentrum* E.B.G. Jones & Abdel-Wahab and *Marinokulati* E.B.G. Jones & K.L. Pang. The three genera *Juncigena*, *Fulvocentrum* (formerly *Swampomyces*), and *Marinokulati* (formerly *Chaetosphaeria*) were originally described from marine habitats in the USA, Egypt, and Bulgaria, respectively [1–4].

Jones et al. [5] placed Juncigenaceae along with Etheirophoraceae, and Torpedosporaceae in Torpedosporales. Two addtional new marine genera *Khaleijomyces* Abdel-Wahab and *Elbamycella* A. Poli, Bovio, Prigione & Varese were introduced to Juncigenaceae from Saudi Arabia and Italy, respectively [6, 7]. The family currently contains five genera and nine species i.e., *Elbamycella rosea*, *Fulvocentrum aegyptiacum*, *F*. *clavatisporum*, *F*. *Rubrum*, *Juncigena adarca*, *J*. *fruticosae*, *Khaleijomyces marinus*, *K*. *umikazeanus*, and *Marinokulati chaetosa* [1, 2, 6–10]. Members of *Juncigenaceae* were mainly reported from intertidal driftwood, submerged sea grasses and brown algae in temperate and subtropical coastal regions worldwide, including Bulgaria, Egypt, Italy, Japan, Saudi Arabia, and the USA [2, 6–11].

The genus *Juncigena* was introduced by Kohlmeyer et al. [2], and was typified by *J*. *adarca* Kohlm., Volkm.-Kohlm. & O.E. Erikss., that was described from senescent leaves of *Juncus roemerianus* Scheele in Atlantic coast, North Carolina, USA. It is characterized by immersed, periphysate ascomata; unbranched pseudoparaphyses attached at both the top and bottom of the ascomatal venter; fusiform to cylindrical, short pedicellate asci with an apical ring; and hyaline, 3-septate ascospores [2]. The asexual morph of *Juncigena adarca* was initially described as *Cirrenalia adarca*, and was characterized by having helicoid, brown conidia with conidial cells that increase in size from the base to the top [2]. Based on phylogenetic and morphological evidence* C*. *adarca* was later transferred to *Moheitospora* as *M*. *adarca* (Kohlm., Volkm.-Kohlm. & O.E. Erikss.) Abdel-Wahab, Abdel-Aziz & Nagah., along with the type species, *M*. *fruticosae* Abdel-Wahab, Abdel-Aziz & Nagah. [8]. *Moheitospora fruticosae* was distributed in marine habitats in Egypt and exhibit coiled conidia with conidial cells that are similar in size and shape, and mature conidia appear to be muriform [8]. Jones et al. [5] synonymized the asexual *C*. *adarca* with its sexual morph *Juncigera adarca*. *Moheitospora fruticosae* was synonymized under *Juncigena* following the one fungus = one name initiative [12]. The two species formerly placed in *Moheitospora* are currently treated as synonyms of *Juncigena*. *Juncigena* was initially classified as a member of the Magnaporthaceae [2, 13]. Then, it was referred to the Hypocreales *incertae sedis* by Jones et al. [14]. The genus assigned at the molecular level to the TBM (*Torpedospora*/*Bertia*/*Melanospora*) clade in Hypocreomycetidae, Sordariomycetes [8, 15]. Later, Jones et al. [5] classified it under Juncigenaceae, Torpedosporales. Presently, the genus included *Juncigena adarca* and *J*. *fruticosae*, both marine saprobes on submerged plant materials [11, 16, 17].

Marine fungi play crucial roles in the decomposition of plant material and nutrient cycling in coastal ecosystems; however, their diversity remains inadequately explored, particularly in the Mediterranean and North African regions. The present study aimed to investigate lignicolous marine fungi from the Egyptian Mediterranean coast and to characterize an undescribed species of *Juncigena* based on detailed morphological comparisons and multi-locus phylogenetic analyses. Detailed morphological illustrations with photomicrographs, a comprehensive description, and multi-locus phylogenetic tree, are presented including appropriate taxonomic justifications to support the delimitation and phylogenetic placement of the new species. This study expands the knowledge of the known species diversity of *Juncigena* and provides new ecological and geographic distribution data, as well as ITS sequence information, thereby addressing a significant taxonomic and geographic gap in the knowledge of marine fungi.

## Materials and methods

### Sample collection, morphological observation and isolation

Decaying intertidal wood samples were collected from El-Shatby beach of the Mediterranean Sea in Alexandria Governorate (31°12′40.3"N 29°54′45.6"E), Egypt, in February 2023. The specimens were incubated in moisture chambers at room temperature (25 °C) and examined periodically over the subsequent three months using an Olympus SZ61 stereomicroscope (Olympus, Tokyo, Japan). Fungal structures were photographed using an Olympus BX51 compound microscope fitted with a Toup Tek XCAM1080PHA (Toup Tek, Zhejiang, China) digital imaging system. Pure cultures of the new collection were obtained by single spore isolation [8]. Colony characters were observed after 4 weeks of growth on glucose, yeast and peptone with agar (GYPA), and malt extract agar (MEA; 2% w/v; Techno Pharmchem, India) medium. To obtain asexual morph, a small piece of pure culture that was grown on potato dextrose agar (PDA; Oxoid, Basingstoke, England) for one month was placed on Sea water agar, MEA, and GYPA, and then sterilized matchsticks were placed on either side of the plate for disturbing the colony and incubated it for 3 months at 25 °C. Fungi growing on matchsticks were incubated in Petri plates lined with sterile, wet filter paper for 3 months with regular observations. Ascomata of the sexual morph were sectioned using a Leica CM 1100 Cryostat (Leica Biosystems, Nussloch, Germany). Voucher specimens and ex-type living culture are preserved in the Sohag University microbial culture collection, Egypt (SUMCC). Nomenclature and description of the new taxon were deposited in MycoBank.

### DNA extraction, sequencing and phylogenetic analysis

DNA was extracted from the pure cultures grown on GYP broth using the QIAamp DNA Mini Kit (Qiagen, Hilden, Germany). The partial nuclear small subunit rDNA (SSU), internal transcribed spacers of rDNA (ITS), and partial nuclear large subunit rDNA (LSU) sequences were amplified with primers sets NS1/NS4, ITS1/ITS4, and LR0R/LR7 respectively [18, 19]. PCR amplification was performed in a 25 µL reaction volume using Solgent EF-Taq as follows: 2.5 µL of 10X EF-Taq buffer, 1 µL of each primer, 1 µL of DNA template, 0.5 µL of 10 mM dNTPs (T), 0.25 µL of EF-Taq (2.5 U), and ddH2O to a final volume of 25 µL. The thermocycling conditions included an initial denaturation at 95 °C for 5 min, 35 cycles of denaturation at 94 °C for 20 s, annealing at 56 °C for 40 s, and extension at 72 °C for 1 min, with a final extension at 72 °C for 5 min. The products were purified and sequenced by Solgent Co., Ltd., (Daejeon, South Korea). New contig sequences were assembled and deposited in GenBank (Table 1). BLASTn searches of newly generated sequences were performed in GenBank. Their closest matches together with sequences selected from recently published data were downloaded to build the final sequence data set [6, 7, 11]. Sequences of SSU, ITS, and LSU regions were separately aligned using Clustal X [36] and optimized manually by trimming terminal bases and ambiguously aligned regions. Pairwise uncorrected (p) distances were calculated in PAUP* v.4.0 [37] using trimmed SSU and LSU rDNA alignments for taxa belonging to the family Juncigenaceae; gaps were treated as missing data.

**Table 1:** Fungal species used for phylogenetic analyses in this study

Species	Strain	GenBank Accession Numbers			References
		**SSU**	**ITS**	**LSU**	
*Chaetopsina aquqtica*	SUMCC H-18001 T	–	MW633072	MW633073	[20]
*Ch. aurantisalinicola*	MFLU 18–0566 T	–	MN047103	MN017868	[21]
*Clonostachys pityrodes*	GJS95-26	AY489696	–	AY489728	[22]
*Cl. rosea*	GJS90-227	AY489684	–	AY489716	[22]
*Cordyceps militaris*	NRRL 28021	AF327392	–	AF327374	[23]
*Elbamycella rosea*	MUT 5443 T	MK775502	MK775497	MK775500	[7]
*E. rosea*	MUT 4937 T	MK775501	MK775496	MK775499	[7]
*Etheirophora blepharospora*	JK5397A	–	–	EF027723	[15]
*Falcocladium multivesiculatum*	CBS 120386	JF831928	JF831936	JF831932	[1]
*Fa*.* sphaeropedunculatum*	CBS 111292	JF831929	JF831938	JF831933	[1]
*Fa. thailandicum*	CBS 121717 T	JF831930	JF831939	JF831934	[1]
*Fa. turbinatum*	BCC 22055^ T^	JF831931	JF831937	JF831935	[1]
*Fulvocentrum aegyptiacum*	CY2973 ^T^	AY858943	–	AY858950	[24]
*Fu. clavatisporium*	LP83	AY858945	–	AY858952	[24]
*Fu. rubrum*	CBS H-22565 T	–	–	MG992006	[9]
*Halosphaeria appendiculata*	NTOU4004	KX686781	–	KX686782	[25]
*Hypocrea lutea*	NBRC 104902	–	JN943364	JN941458	[26]
*Juncigena adarca*	JK5548A	EF027720	–	EF027727	[15]
*J. adarca*	JK5235A	EF027719	–	EF027726	[15]
***J. alexandrina***	**SUMCC 23002**	**PX630356**	**PX630355**	**PX630354**	**This study**
*J. fruticosae*	EF14^T^	GU252146	–	GU252145	[8]
*Khaleijomyces marinus*	CBS H-22564 T	MG717679	–	MG717678	[6]
*K. marinus*	MD 1348	–	–	MG717677	[6]
*K. umikazeanus*	NBRC 105287 T	MN921252	–	MN921253	[10]
*Lignincola laevis*	JK 5180A	U46873	JQ838037	U46890	[27]
*Marinokulati chaetosa*	BCRC FU30271	KJ866929	–	KJ866931	[1]
*M. chaetosa*	BCRC FU30272	KJ866930	–	KJ866932	[1]
*Nohea umiumi*	NTOU4006	KX686795	–	KX686796	[25]
*Petriella setifera*	AFTOL 956	DQ471020	–	DQ470969	[28]
*Pileomyces formosanus*	BBH30192	KX686803	JX003862	KX686804	[25]
*Qarounispora grandiappendiculata*	SUMCC H-17009 T	OK043819	OK042831	OK043820	[29]
*Safagamyces marinus*	SUMCC H-20001^ T^	ON244698	–	ON244695	[30]
*Scopulariopsis brevicaulis*	G415	KJ443075	KJ443250	KJ443119	[31]
*Swampomyces armeniacus*	JK5059C	EF027721	–	EF027728	[15]
*S*.* triseptatus*	CY2802	AY858942	–	AY858953	[24]
*Trichoderma viride*	GJS89-127	AY489694	–	AY489726	[22]
*Stilbohypoxylon elaeidis*	MFLUCC 15-0295b	MT495461	MT496746	MT496756	[32]
*Torpedospora ambispinosa*	MUT 3537^ T^	MK775498	MK775503	MK775495	[7]
*T*.* mangrovei*	NBRC 105264^ T^	GU252150	LC146768	GU252149	[8]
*T*. *radiata*	BCC11269	AY858938	–	AY858948	[24]
*T. radiata*	AFTOL 751	DQ470999	–	DQ470951	[15]
*T. yanbuensis*	SUMCC H-11002^ T^	ON595716	–	ON595744	[33]
*Xylaria acuta*	AFTOL 63	AY544719	–	AY544676	[34]
*X. hypoxylon*	AFTOL 51	AY544692	DQ491487	AY544648	[34, 35]

Newly generated sequences are indicated in bold; T signifies ex-type strains; – indicates unavailable data in GenBank

Maximum likelihood (ML), maximum parsimony (MP), and Bayesian inference (BI) phylogenetic analyses were performed for each of the single-locus and multi-locus alignments. Maximum likelihood analysis was conducted by RAxMLGUI v. 2.0.13 [38] under GTR + GAMMA substitution model with 1,000 bootstrap iterations was set. Maximum-parsimony analyses were conducted in PAUP* v.4.0 with the heuristic search option with 1000 random sequence additions and tree bisection reconnection (TBR) branch swapping. Bayesian inference posterior probabilities (BYPP) were evaluated in MrBayes v. 3.1.2 [39], with tree sampling every 1000 generations during the 4,000,000 generations run of four simultaneous Markov chains. MrModeltest v.2.3 [40] was used to select the best-fitting substitution model for Bayesian inference of the combined SSU, ITS, and LSU datasets, with SYM + G identified as the optimal model under the Akaike Information Criterion (AIC). Other details are as provided by by Bakhit and Abdel-Wahab [30, 41]. Obtained phylogenetic tree was represented by NJplot [42] and then edited using Adobe Illustrator CC (Adobe Systems Inc., CA, USA). Bootstrap support values ≥ 70% are indicated at the tree nodes, and thickened branches correspond to Bayesian posterior probabilities (BYPP) ≥ 0.90.

## Results

### Phylogenetic analysis

**Fig. 1:**
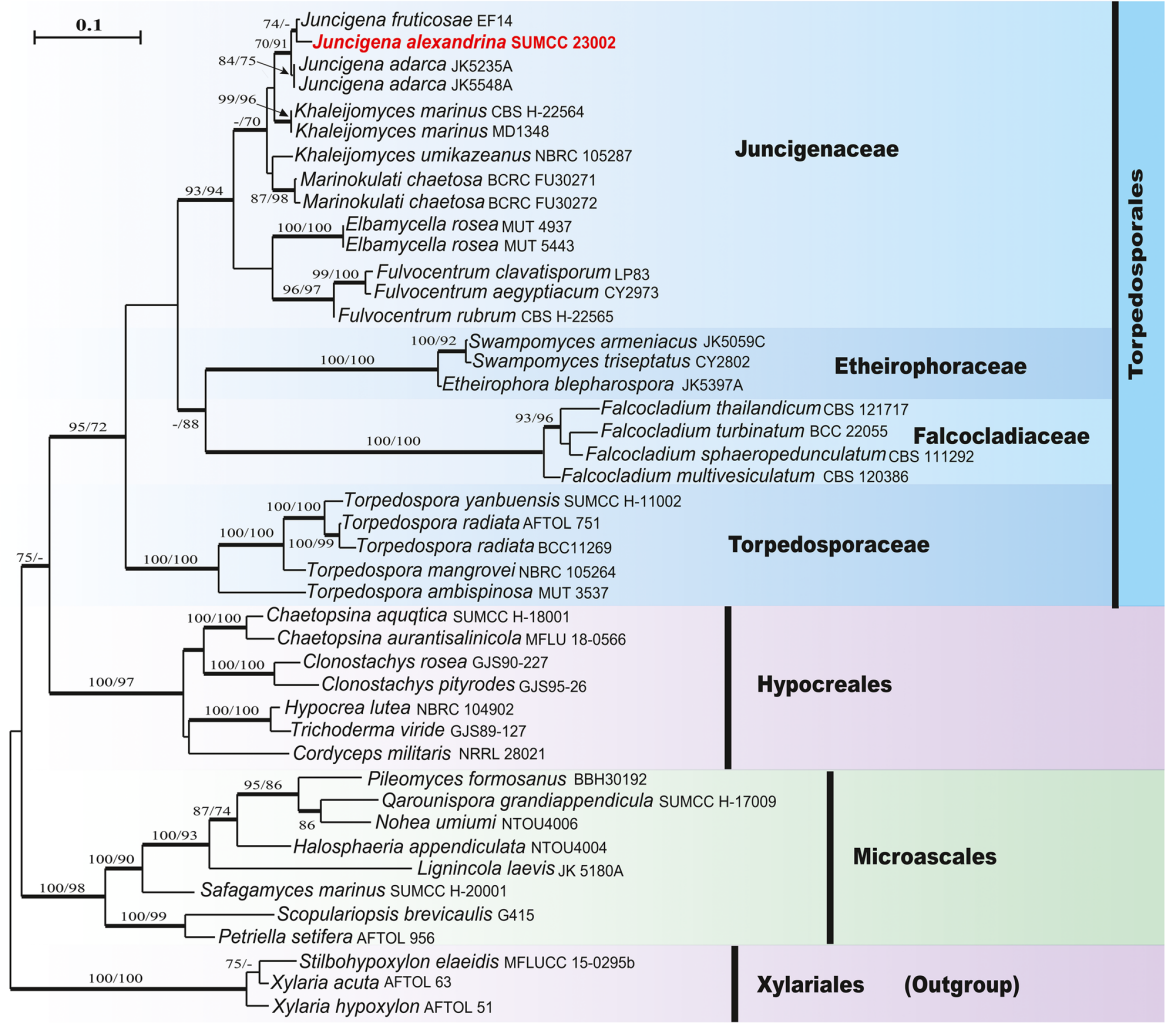
Phylogenetic tree generated from ML analysis (RAxML) based on a combined SSU, ITS, and LSU sequences for *Juncigena* with other genera in Juncigenaceae and related orders. ML and MP bootstrap supports (≥ 70%) are indicated around the nodes. Branches received Bayesian pp ≥ 0.90 are in bold. The newly generated sequences are indicated in red

The combined SSU, ITS, and LSU sequence dataset comprised 44 taxa, with *Stilbohypoxylon elaeidis* (MFLUCC 15-0295b), *Xylaria acuta* (AFTOL 63), and *Xylaria hypoxylon* (AFTOL 51) as outgroup taxa. The MP dataset consisted of 2,719 characters (SSU = 1250, ITS = 617, LSU = 852) after alignment that included 1,668 constant, 310 variable parsimony-uninformative and 741 parsimony-informative characters. The most parsimonious tree was with a tree length of 1,734 steps, a consistency index (CI) of 0.5415, a rescaled consistency index (RC) of 0.4120 and a retention index (RI) of 0.7609. The ML analysis of the combined dataset yielded the best-scoring tree (Fig. 1) with a final ML optimization likelihood value of −15,879.403943. The matrix had 1,037 distinct patterns with 41.04% undetermined characters or gaps. Estimated base frequencies were A = 0.236801, C = 0.264393, G = 0.290371, T = 0.208435; substitution rates, AC 0.919463, AG = 2.239539, AT = 1.572438, CG = 0.862093, CT = 5.829662, GT = 1.0; gamma distribution shape parameter α = 0.494733. The Bayesian analysis resulted in 40,000 trees after four million generations. The first 25% of the generated trees were discarded as burn-in, while the remaining trees were used to calculate posterior probabilities. The topologies obtained from the three analyses were similar. Phylogenetic analyses of the combined SSU, ITS, and LSU sequence data placed the new taxon, *Juncigena alexandrina* (SUMCC 23003) within *Juncigena* but distinct from previously described species (Fig. 1). Single-locus SSU and LSU datasets were analyzed to compare tree topology and clade stability relative to the combined analysis, and no significant differences were observed in the LSU-based phylogeny. In the SSU rDNA tree, the new taxon clustered with *Fulvocentrum aegyptiacum* and *F. clavatisporum* within Juncigenaceae. The resulting trees are shown in the supplementary material (Figures S1–S2).

#### Taxonomy

**Fig. 2:**
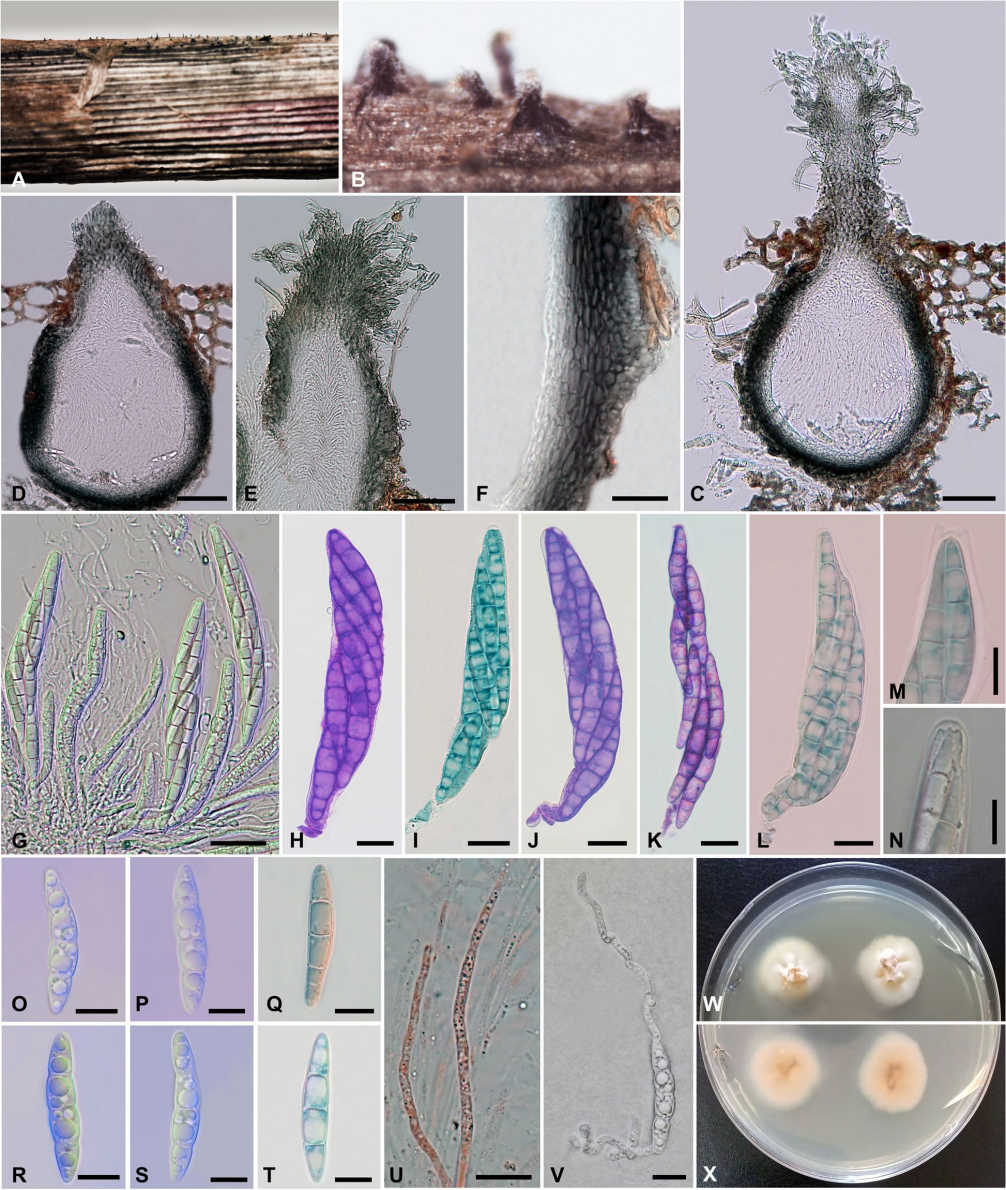
*Juncigena alexandrina* (SUMCC H-23002, holotype). **A**, **B** Appearance of ascomata on host surface. **C**, **D** Sections through ascomata. **E** Section through ostiolar neck with periphyses and hairs. **F** Peridium. **G**–**L** Asci; **H**–**L** stained in toluidine blue. **M**, **N** Close up of ascal tips. **O**–**T** Septate ascospores with guttules. **U** Pseudoparaphyses stained in Congo red. **V** A germinated ascospore. **W**, **X** Colonies on GYPA, above (**W**) and below (**X**). Scale bars: C, D, E = 50 μm, F, G = 20 μm, H-V = 10 μm

*Juncigena alexandrina* Bakhit sp. nov. – Fig. 2.

MycoBank no.: MB861485.

Etymology. In reference to the the city name, Alexandria, where the holotype was collected.

GenBank accession no.: SSU: PX630356; ITS: PX630355; LSU: PX630354.

Saprobic on a decaying stem of *Phragmites australis* (Cav.) Trin. ex Steud. Sexual morph: Ascomata 215–305 μm high, 190–250 μm diam. (x̄ = 253 × 211 μm, n = 12), solitary, scattered, semi-immersed to erumpent, perithecial, subglobose to pyriform, papillate, black, coriaceous, uni-loculate, central ostiolate with a long neck, ascospores ooze from the ostiole forming hyaline to pale yellow spore mass. Ostiolar neck 140–255 μm long, 55–100 μm wide (x̄ = 203 × 78 μm, *n* = 9), straight, cylindrical to subcylindrical, dark grey to black, with hyaline periphyses, and covered with long, 3–6 μm wide, branched, septate, stright or flexuous hairs with dark brwon to hyaline tips. Peridium 28–44 μm wide (x̄ = 36 μm, n = 9), with distinct two layers; with 9–12 cell layers, outer layer composed of thin-walled, carbonaceous, irregular, *textura prismatica* or flattened cells of *textura angularis*, inner layer composed hyaline, thick-walled, flattened cells of *textura angularis*. Hamathecium comprises 110–170 μm high, 1–2 μm wide, hyaline, branched, septate pseudoparaphyses. Asci 100–135 × 11–17.5 μm (x̄ = 116 × 15 μm, n = 38), 8-spored, unitunicate, cylindrical to clavate, straight to slightly curved, short pedicellate, apically rounded, with a J −, apical ring. Ascospores 34–45 × 5.5–8 μm (x̄ = 40 × 7 μm, n = 61), overlapping 2–3-seriate, hyaline, thick-walled, smooth-walled, fusiform with rounded ends, 3-transverse septate, slightly constricted at septa, broader towards the center and straight to slightly curved, multiguttulate. Asexual morph: Undetermined.

##### Cultural characteristics

Ascospores germinated on 50% sea water agar within 24 h with germ tubes developed from both ends. Colonies on GYPA medium reached 25–30 mm diam., after 4 weeks at 25 °C, circular, center raised, with abundant hyphal tufts, margin regular, with sparse aerial mycelium, white on the surface, creamy to pale yellow in reverse, with hyaline, septate, branched, 2.5–5 μm wide mycelium, and sometimes grouped in little tufts. Colonies on MEA medium reach 22–26 mm in 4 weeks, white on the surface, pale yellow in the reverse.

##### Material examined

EGYPT, Alexandria Governorate, El-Shatby beach of the Mediterranean Sea, 31°12′40.3"N 29°54′45.6"E, on a decaying stem of *Phragmites australis* (Poaceae), 23 February 2023, M.S. Bakhit (SUMCC H-23002; holotype), ex-type living culture SUMCC 23003.

#### Notes

*Juncigena alexandrina* is introduced as a new species based on morphology and phylogenetic analysis of combined SSU, ITS, and LSU sequence datasets (Fig. 1). Molecular phylogenetic analyses placed the new taxon, *J. alexandrina* (SUMCC 23002) in a clade containing the two marine *Juncigena* species, *J. adarca* (JK5548A and JK5235A) and *J. fruticosae* (EF14), by forming a sister lineage with *J. Fruticosae* and having 74% bootstrap support in ML analysis. *Juncigena alexandrina* (SUMCC H-23002) morphologically resembles the generic type, *J. adarca*, in having subglobose to pyriform, periphysate ascomata; 8-spored, unitunicate, short pedicellate, asci with an apical ring; and hyaline, 3-septate ascospores [2, 43]. However, *Juncigena alexandrina* has wider, semi-immersed to erumpent ascomata (190–250 μm in diam.) with a neck (140–255 μm long), whereas *J. adarca* has completely submersed ascomata under the cortex (135–200 µm in diam.) with a shorter neck (85–170 µm long) [2]. The osliolar neck in *J. alexandrina* covered with long hairs, that are branched, septate, stright or flexuous and dark-brown with hyaline tips. *Juncigena adarca* has completely submerged ascomata without hairs covering the ostiolar neck [5]. Peridial wall in *J. adarca* is 10–20 μm wide and consists of 8–10 layers of thick-walled ellipsoidal to subglobose cells, forming a *textura angularis*, whereas in *J. alexandrina*, it is 28–44 μm wide and with 9–12 cell layers of *textura prismatica* or flattened cells of *textura angularis*. Moreover, the asci of *J. alexandrina* are cylindrical to clavate, straight to slightly curved, and wider than those of *J. adarca* (11–17.5 vs. 10–13 μm). Asci in *J. adarca* are fusiform to cylindrical with wall refractive at the apex [2]. Ascospores of *J. alexandrina* are overlapping 2–3-seriate, fusiform with rounded ends, straight to slightly curved, broader at the center, and larger than those in *J. adarca* (34–45 × 5.5–8 μm vs. 26.5–34.5 × 6–7 µm). Ascospores of *J. adarca* are 1–2-seriate, fusiform to elongate ellipsoidal and second cell from the top usually widest [2, 43]. Currently, this genus includes two species: *J. adarca* and *J. fruticosae*, with the sexual morph observed only in the former. Asexual morphs in the two species are chrachterized by having brown and septate helicoid conidia [2, 8]. Asexual morph of *J. alexandrina* was not observed in both natural substrate and after four months of incubation of the fungus on matchsticks in Petri plates.

Pairwise uncorrected (p) distance analyses based on LSU sequences show that *Juncigena alexandrina* (SUMCC 23003) differs from *J. fruticosae* (EF14) by 0.5% and from *J. adarca* (JK5548A) by 0.7%, whereas no LSU divergence was detected between the two available isolates of *J. adarca* (JK5548A and JK5235A). Similarly, SSU-based p-distance analyses indicate that *Juncigena alexandrina* differs from *J. fruticosae* by 0.8% and from *J. adarca* by 0.9%, while the p-distance between *J. adarca* and *J. fruticosae* is only 0.1% (Table 2). ITS sequences are unavailable for both *J. fruticosae* and *J. adarca*. Therefore, *J*. *alexandrina* is introduced here as a new species based on morphology and molecular evidence.

**Table 2:**
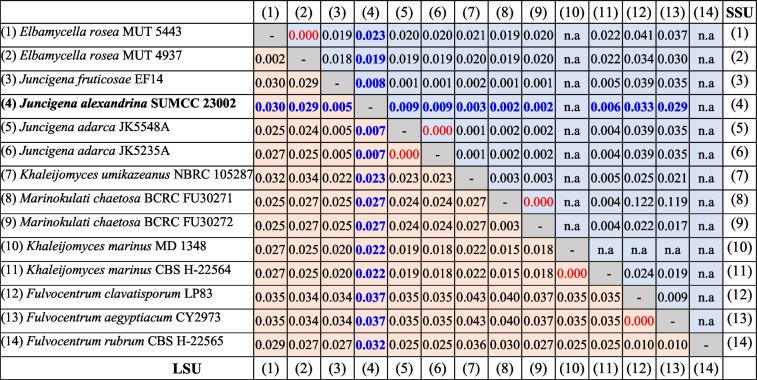
Pairwise p-distance values for aligned LSU (lower left triangle) and SSU (upper right) gene sequences

n.a. = sequence data not available, Data for *Juncigena alexandrina *in blue, divergence values = zero in red

## Discussion

The first report of marine fungi from Egypt was *Pythium marinum*, infesting *Porphyra leucosticta* in the Mediterranean Sea by Aleem [44]. Later, Schatz [45] recorded *Adomia avicenniae* from pneumatophores of *Avicennia marina* from both Australia and Egypt. Subsequently, studies of marine fungi colonizing decaying plant debris in Egypt have significantly advanced in the last 25-years. Six new genera and ten new fungal species have been introduced as new to science from marine environments in Egypt [1, 3, 8, 12, 29, 41, 46, 47].

The Egyptian Mediterranean coastline extends for about 1,050 km and holds significant environmental importance. Abdel-Wahab et al. [47] discovered two new *Corollospora* species, *C*. *anglusa* Abdel-Wahab & Nagah. and *C*. *Portsaidica* Abdel-Wahab & Nagah. from the coast of the Mediterranean Sea in Egypt. Abdel-Aziz [48] recorded 31 marine fungi from 100 submerged driftwood samples collected from two sandy beaches along the Mediterranean coast of Egypt. *Corollospora maritima* Werderm., was the most common fungus found in her study. Abdel-Wahab et al. [8] described three new asexual genera and species from the Mediterranean Sea coast of Egypt: *Halazoon* (*H*. *melhae*), *Moleospora* (*M*. *maritima*), and *Moheitospora* (*M*. *fruticosae*). Later, Réblová et al. [12] transferred *M*. *fruticosae* to the genus *Juncigena* as *J*. *fruticosae*.

Single-collection descriptions are not uncommon for intertidal marine ascomycetes owing to the scarcity and transience of suitable substrates; where congruent morphological and phylogenetic evidence is available, such taxa are provisionally accepted pending further collections. In this article, a new species, *Juncigena alexandrina* was introduced from a decaying stem of *Phragmites australis* collected from El-Shatby beach of the Mediterranean Sea in Alexandria. Multi-locus analyses of the combined SSU, ITS, and LSU rDNA sequence data placed the new taxon within *Juncigena* as a phylogenetically distinct species in a clade containing *J. adarca* and *J*. *fruticosae*. All morphological measurements and molecular data for *Juncigena alexandrina* are currently derived from a single collection (SUMCC H-23002) and its ex-type culture (SUMCC 23003); future sampling will be necessary to assess intraspecific variation in both morphology and DNA barcode regions.

Pairwise uncorrected (p) distance matrices are widely applied in fungal taxonomy to quantify sequence divergence and support species delimitation. Analyses based on LSU and SSU rDNA sequences revealed low to moderate interspecific divergence within Juncigenaceae, with LSU distances ranging from approximately 0.5–3.7% and SSU distances from 0.1–4.1%, whereas intraspecific variation was negligible or absent (0.0–0.3%). Notably, *Juncigena alexandrina* exhibits clear genetic divergence from *J. adarca* and *J. fruticosae*, corroborating its distinct phylogenetic placement inferred from combined rDNA analyses.

ITS sequence data are currently unavailable for *Juncigena adarca* and *J. fruticosae*, limiting species-level phylogenetic resolution within the genus and necessitating reliance on the more conserved LSU and SSU loci. In contrast, the inclusion of ITS data for the newly described species provides additional phylogenetically informative characters that strengthen its taxonomic delimitation and highlights the need for expanded ITS sequencing in *Juncigena*.

Future studies incorporating additional isolates from the type locality and nearby regions, along with expanded multi-locus datasets, will be essential to further evaluate intraspecific variation and confirm species boundaries within *Juncigena*.

The present study highlights the importance of investigating sexual–asexual relationships in fungal species, as taxa historically described under separate names based on different life stages have been unified through molecular evidence, in accordance with the “one fungus = one name” principle, resulting in a more stable and coherent taxonomy.

The new taxon was recorded on a decaying stem of *Phragmites australis* collected from marine habitat in Egypt. This represents the first record of a *Juncigena* species colonizing decaying stems of *Phragmites australis*. *Juncigena* species are known exclusively as marine saprobes [2, 8], this study]. A high fungal diversity is known from *Phragmites australis* in both estuarine and freshwater habitats in Egypt [41, 49–51].

## Conclusion

The family Juncigenaceae currently comprises nine species, including five—*Fulvocentrum aegyptiacum*, *F*. *clavatisporum*, *F*. *Rubrum*, *Juncigena fruticosae*, *Khaleijomyces marinus* were discovered from marine environments in Egypt and Saudi Arabia. This study introduces, based on a single collection and ex-type culture, a novel fungus, *Juncigena alexandrina*, from submerged decaying stems of *Phragmites australis* in the Mediterranean Sea (Alexandria Governorate, Egypt), identified by congruent morphological differences and rDNA-based phylogenetic placement; future sampling will test the stability of these diagnostic features across additional isolates. This represents the first report of a *Juncigena* species colonizing *P*. *australis*, expanding the known substrate range of the genus. This finding provides a significant update to the global inventory of this family, enhancing our understanding of fungal diversity in the region. Ultimately, this research emphasizes the importance of integrating conventional approaches with molecular methods in fungal taxonomy, supporting global efforts to document and conserve fungal diversity.

## Supplementary Information

Supplementary Material 1

## Data Availability

The new taxon was registered in Mycobank as MB861485. Sequences generated during this study were deposited in NCBI database with the accession numbers PX630356, PX630355and PX630354.
