# Supplementary material for: Introducing Juncigena alexandrina sp. nov. (Ascomycota, Juncigenaceae) from the Mediterranean coast of Egypt, based on morphology and multi-locus phylogeny
Source: BMC Microbiol. 2026 Mar 7;26:322. doi: 10.1186/s12866-026-04825-y (PMC13063913; doi:10.1186/s12866-026-04825-y)
Supplement: Supplementary file 1 — Supplementary Material 1 [file 12866_2026_4825_MOESM1_ESM.docx]

**Introducing *Juncigena alexandrina* sp. nov.** **(Ascomycota, Juncigenaceae) from the Mediterranean coast of Egypt, based on morphology and multi-locus phylogeny**

**Supplementary material**

**
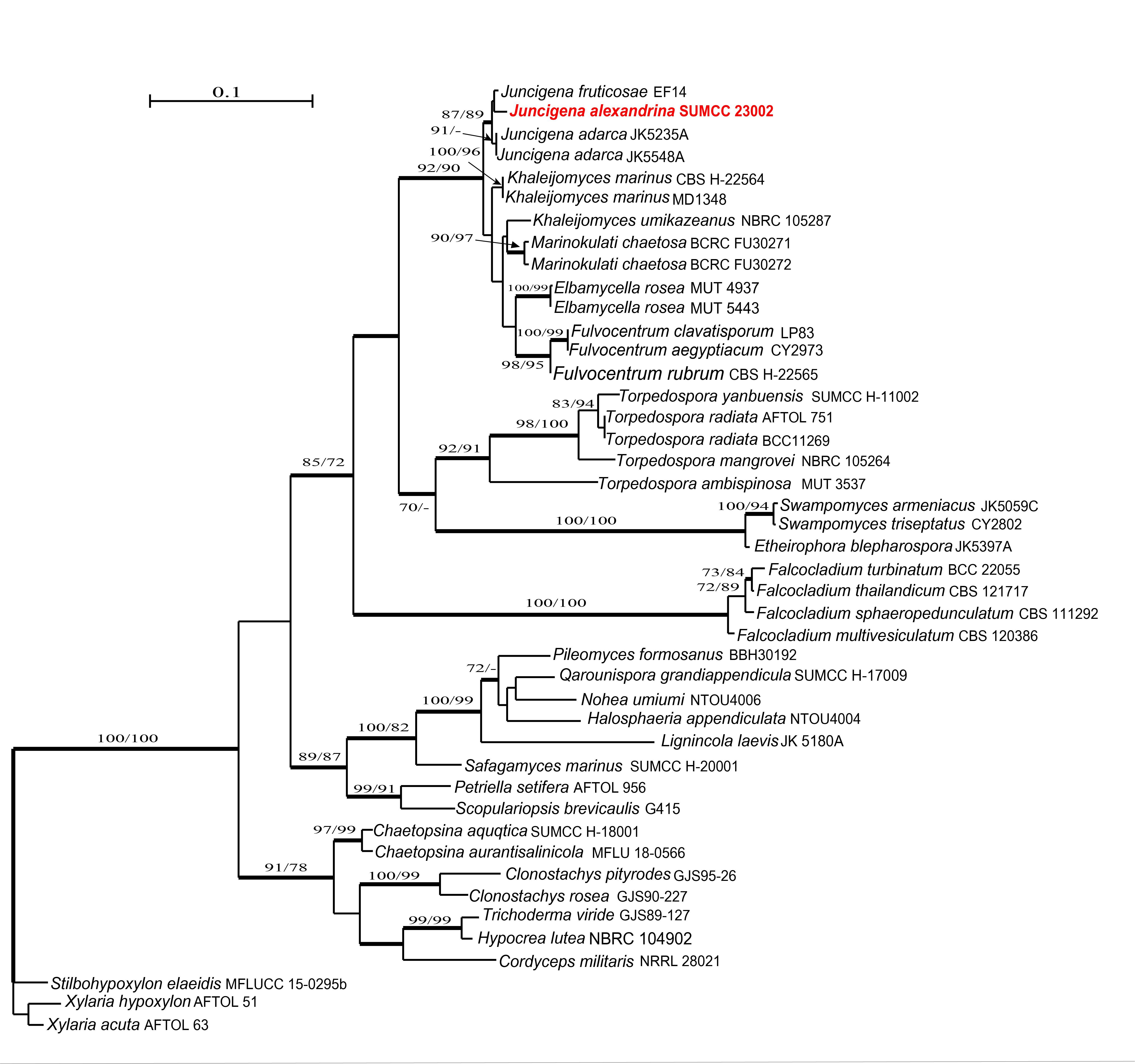
**

**Fig. S1** Phylogenetic tree generated from ML analysis (RAxML) based on LSU sequence for *Juncigena* with other genera in Juncigenaceae and related orders. ML and MP bootstrap supports (≥70 %) are indicated around the nodes. Branches received Bayesian pp ≥ 0.90 are in bold. The newly generated sequences are indicated in red.


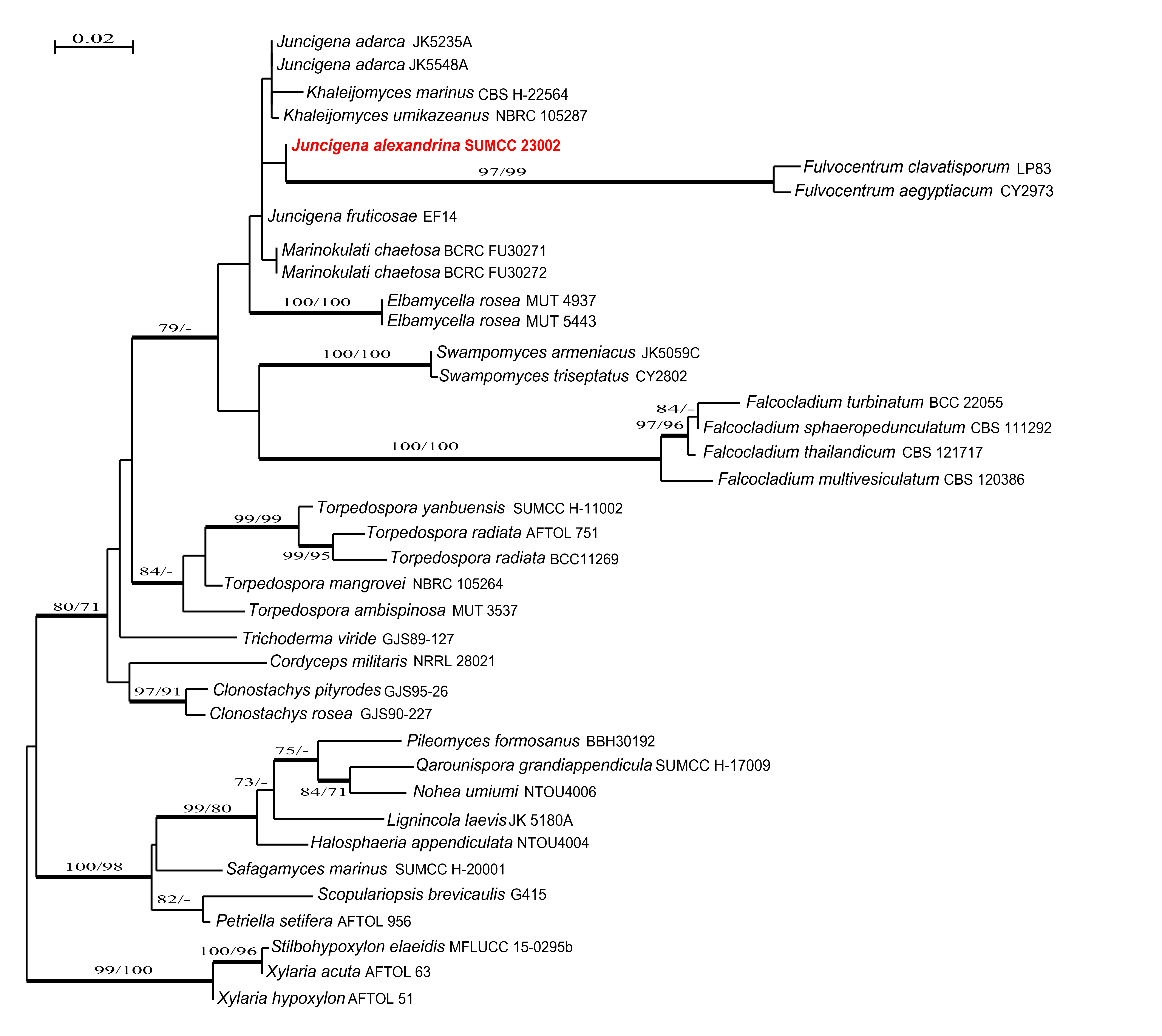


**Fig. S2** Phylogenetic tree generated from ML analysis (RAxML) based on SSU sequence for *Juncigena* with other genera in Juncigenaceae and related orders. ML and MP bootstrap supports (≥70 %) are indicated around the nodes. Branches received Bayesian pp ≥ 0.90 are in bold. The newly generated sequences are indicated in red.
